# Early gene expression divergence between allopatric populations of the house mouse (*Mus musculus domesticus*)

**DOI:** 10.1002/ece3.447

**Published:** 2013-01-31

**Authors:** Jarosław Bryk, Mehmet Somel, Anna Lorenc, Meike Teschke

**Affiliations:** 1 Max Planck Institute for Evolutionary BiologyAugust–Thienemann–Str. 2, 24306, Plön, Germany; 2 Partner Institute for Computational Biology, Shanghai Institutes for Biological Sciences, Chinese Academy of Sciences200031, Shanghai, China; 3 Max Planck Institute for Evolutionary Anthropology04103, Leipzig, Germany

**Keywords:** Evolution, gene expression, population divergence, wild mice

## Abstract

Divergence of gene expression is known to contribute to the differentiation and separation of populations and species, although the dynamics of this process in early stages of population divergence remains unclear. We analyzed gene expression differences in three organs (brain, liver, and testis) between two natural populations of *Mus musculus domesticus* that have been separated for at most 3000 years. We used two different microarray platforms to corroborate the results at a large scale and identified hundreds of genes with significant expression differences between the populations. We find that although the three tissues have similar number of differentially expressed genes, brain and liver have more tissue–specific genes than testis. Most genes show changes in a single tissue only, even when expressed in all tissues, supporting the notion that tissue–specific enhancers act as separable targets of evolution. In terms of functional categories, in brain and to a smaller extent in liver, we find transcription factors and their targets to be particularly variable between populations, similar to previous findings in primates. Testis, however, has a different set of differently expressed genes, both with respect to functional categories and overall correlation with the other tissues, the latter indicating that gene expression divergence of potential importance might be present in other datasets where no differences in fraction of differentially expressed genes were reported. Our results show that a significant amount of gene expression divergence quickly accumulates between allopatric populations.

## Background

Gene expression changes contribute significantly to the evolutionary divergence of populations and species (King and Wilson 1975; Wray 2007), but there is an ongoing debate on how much this is due to neutral divergence versus adaptive changes (Khaitovich et al. 2004, 2005; Hoekstra and Coyne 2007; Carroll 2008; Staubach et al. 2010). For deer mice, it was possible to show that a change in Agouti expression is linked to an adaptive change in coat color and has arisen within a time scale of a few thousand years (Linnen et al. 2009). Fraser et al. (2011) have devised a test for studying cis–regulatory evolution among house mouse subspecies and suggest that over 100 genes may have been subject to lineage–specific regulatory selection. Large–scale changes in gene expression among species, such as humans and chimpanzees (Khaitovich et al. 2005), among mammals (Brawand et al. 2011), or *Mus musculus* and *Mus spretus* (Voolstra et al. 2007), have also been well documented. An interesting observation in the latter study was a striking difference in gene expression divergence among tissues when analyzed between species versus subspecies. While most studies so far have shown that genes expressed in the testis show the highest rate of between–species divergence when compared to other tissues, Voolstra et al. (2007) found that this is not the case when subspecies of the house mouse are compared: the significant changes in testis–expressed genes were less frequent than those in liver or brain. This observation suggests that gene expression differences should be studied at different scales of lineage divergence to gain a deeper insight into their evolutionary dynamics and motivated us to analyze gene expression divergence at the early stage of population differentiation.

## Results

**Figure 1:**
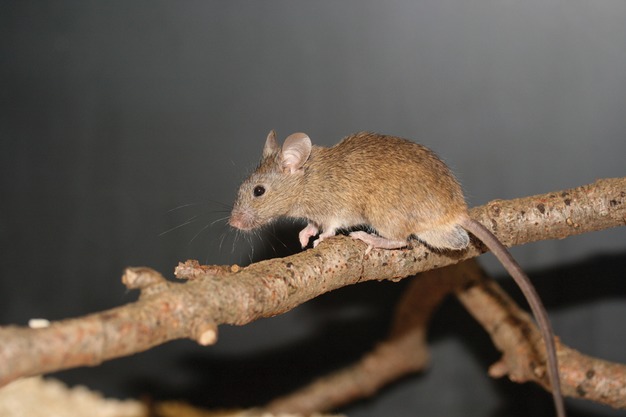
Wild male *Mus musculus domesticus* from the German population. Photograph by Christine Pfeifle.

Animals used in this study (Fig. 1.) were captured in houses, barns or stables in the rural region of Massif Central, in a 20 × 20 km area around and in the town of Severac-le-Chateau in France (44°15′N–44°30′N, 2°45′E–3°15′E) and in the 40 × 70 km area around the cities of Cologne and Bonn in Germany (50°45′N–51°N, 6°45′E–7°E). Sampling sites were at least 300 m apart and only a single pair of animals (a male and a female) was collected from any given sampling site to ensure that the mice from different sites were not related (Ihle et al. 2006). Mice were brought to the lab where they were kept under laboratory conditions in standard cages equipped with housing material, plastic or paper houses and spinning wheels and fed ad libitum. Total RNA from whole brain, liver, and testis from six of the wild–caught males from each of the populations was used for the gene expression analysis. To identify the most reliable signals in the samples, we used two independent microarray technologies (Affymetrix Mouse Genome 430_2 and Agilent Mouse Genome 4 × 44k arrays) to select a common set of genes interrogated by both platforms.

### Identification of expressed genes

After 39,000 Affymetrix probesets were collapsed into 17,144 genes (Dai et al. 2005) and a fraction of genes with no or limited variation in gene expression levels as measured across all samples from both populations was removed using approach recommended by Hahne et al. (2008) (see Methods), we obtained a list of 13,725 genes expressed in any of the three tissues. 10,160, 8751, and 9745 of these genes were expressed in brain, liver, and testis, respectively. The same RNA samples were subsequently run on the Agilent platform. Of over 44,000 Agilent probes, only those that passed preprocessing filtering and hybridized to genes included in the Affymetrix analysis were used in the subsequent analysis (see Methods). We thus obtained a common set of 12,977 genes expressed in any of the tissues and interrogated by both microarray platforms; 9730 of the genes were expressed in brain, 8272 in liver and 9244 in testis, and 5623 were expressed in all three tissues.

### Identification of differently expressed genes

We identified differently expressed genes between the populations by performing a moderated t-test (Smyth 2004) on genes that fulfilled the test's assumption of equal variance between compared groups, and a Mann–Whitney U test for the remaining genes, using a cutoff *P*-value of 0.01 and requiring the same direction of change in both platforms. We used Levene's test to check for equal variance between the populations for each gene or probe in each platform separately, and identified 1292 genes (13.3%) in brain, 1595 (19.3%) in liver, and 1660 (17.8%) in testis on Affymetrix samples, and 859 (8.8%), 694 (8.4%), 957 (10.4%), respectively, on Agilent, that had significantly different variance between the groups (Levene's test, *P* < 0.05). The genes with unequal variance between the populations were largely nonoverlapping between the platforms, with only 171 genes having unequal variance on both Affymetrix and Agilent platforms in brain, 229 in liver and 282 in testis. Overall distributions of variances in each population and tissue were not significantly different on either platform (Levene's test, smallest *P* > 0.14).

Genes that were significantly differently expressed in opposite directions on the two platforms (*P* < 0.01; *n* = 7 in brain, *n* = 2 in liver and *n* = 6 in testis) were removed from further analyses, leaving a total number of genes investigated in each tissue to be 9723 in brain, 8270 in liver and 9238 in testis, 12,975 genes expressed in any tissue and 5614 genes expressed in all three tissues.

Overall, we identified 746 differently expressed genes (the list of genes is provided in supplementary Table 1), with a similar fraction in each tissue: 269 (2.77%) of such genes in brain, 320 (3.87%) in liver and 236 (2.6%) in testis. If one would consider each of the platforms alone, one would find larger numbers of differently expressed genes for each of them, but comparably distributed across tissues (Table 1).

**Table 1:** Number of differentially expressed genes in the common set of genes and in each platform separately

Common set of genes	Both *P* < 0.01 and same direction of change	Affymetrix *P* < 0.01	Agilent *P* < 0.01
Brain (*n* = 9723)	269 (2.8%)	787	887
Liver (*n* = 8270)	320 (3.9%)	620	974
Testis (*n* = 9238)	236 (2.6%)	638	822

We assessed the reliability of the identification of differently expressed genes by estimating what fraction of the observed differently expressed genes could be due to false positives. We performed Levene's test on all 924 possible combinations (462 unique combinations) of sample population names followed by the moderated *t*-test or Mann–Whitney *U-*test for the appropriate genes in each permutation. Median numbers of differently expressed genes (*P* < 0.01) in these permutations were only 3 in brain, 6.5 in liver, and 5 in testis, corresponding to false discovery rates of 1.1%, 2.0%, and 2.1%, respectively. These results indicate that the false positive rate is low.

### Correlations of the microarray platforms

Low overlap of the genes with unequal variance between the two populations, as well as low overlap of differentially expressed genes between the platforms (Table 1.) suggested general limited agreement between the platforms. To assess in more detail how well the two platforms are correlated in their ability to detect different gene expression between the two populations, we used moderated t statistic (Smyth 2004) for the genes with equal variances in each platform to calculate Spearman's rank correlation coefficient, as the t statistic provides information about both the direction and degree of difference between the groups. For the genes from the common set with equal variance between the populations on both platforms, we found correlations of only ρ = 0.55 (*n* = 7744) in brain, 0.67 (*n* = 6212) in liver, and 0.52 (*n* = 6903) in testis. When we restricted the above comparison to genes that are significantly differently expressed between the populations, we found correlations of ρ = 0.84 in brain (*n* = 208), 0.87 in liver (*n* = 252), and 0.86 in testis (*n* = 167), suggesting a smaller influence of technical variation in sample labeling and probe hybridization on expression differences. The incomplete correlations do not apparently result in systematic errors, as in comparisons of fractions of differently expressed genes and their tissue specificity each of the platforms separately provided similar results as the combined dataset (see Table 1 and Fig. 3A–C). However, the genes within each of these comparisons on each platform were not identical, and therefore, we limit most of the analyses to the actual overlap of genes identified in both platforms.

### Tissue correlations

**Figure 2:**
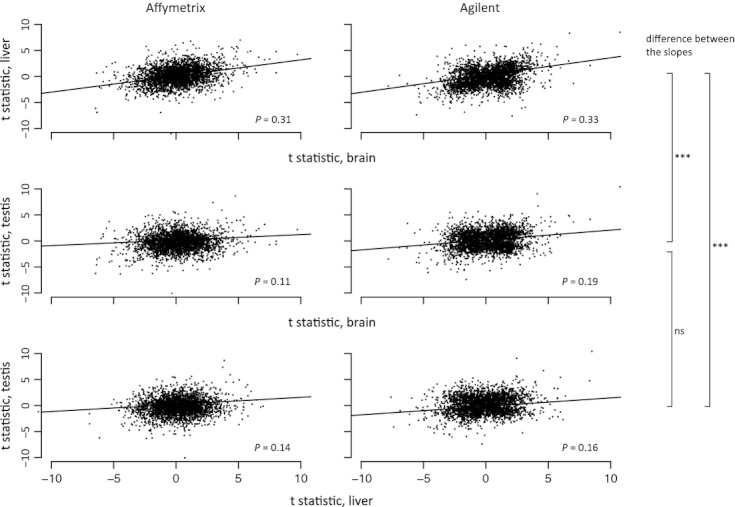
Spearman's rank correlations of t statistics between pairs of tissues from both populations on both platforms for 2762 genes with equal variance between the populations in both platforms and expressed in all tissues in both platforms. Outliers beyond t statistic smaller than −10 and bigger than 10 were omitted from the graphs. Significance values (test for equality of regression slopes (Dalgaard 2008; Zar 2010)): brain–liver vs brain–testis pAffymetrix = 6.05 × 10^−12^ and pAgilent = 3.34 × 10^−7^; brain–liver vs liver–testis pAffymetrix = 1.41 × 10^−9^ and pAgilent=1.14 × 10^−9^; brain–testis vs liver–testis pAffymetrix = 0.23 and pAgilent = 0.25.

To assess whether the genes showing large differences in one tissue also tend to show similar differences in other tissues, we compared the correlations of the t statistics between all three pairs of tissues for the 2762 genes expressed in both platforms in all tissues and with equal variance in all tissues using Spearman' rank correlation coefficient. We found that testis' t-statistics values were consistently least correlated with other tissues, whereas brain and liver showed approximately twice as strong a correlation with each other (Fig. 2). The differences in correlations among pairs of tissues were highly significant for the comparison between brain–liver versus brain–testis (test for equality of regression slopes (Dalgaard 2008; Zar 2010); pAffymetrix = 6.05 × 10^−12^ and pAgilent = 3.34 × 10^−7^), brain–liver versus liver–testis (pAffymetrix = 1,41 × 10^−9^ and pAgilent = 1.14 × 10^−9^), but not significant for the comparison between brain–testis versus liver–testis (pAffymetrix = 0.23 and pAgilent = 0.25). These results hold when we relax the requirement of equal variance between the genes and take all 5614 genes expressed in all tissues in both platforms (data not shown). This suggests that testis diverges in a different way from the other two tissues, although it does not stand out when considering the fraction of differently expressed genes (Table 1).

### Tissue–specific gene expression divergence

**Figure 3:**
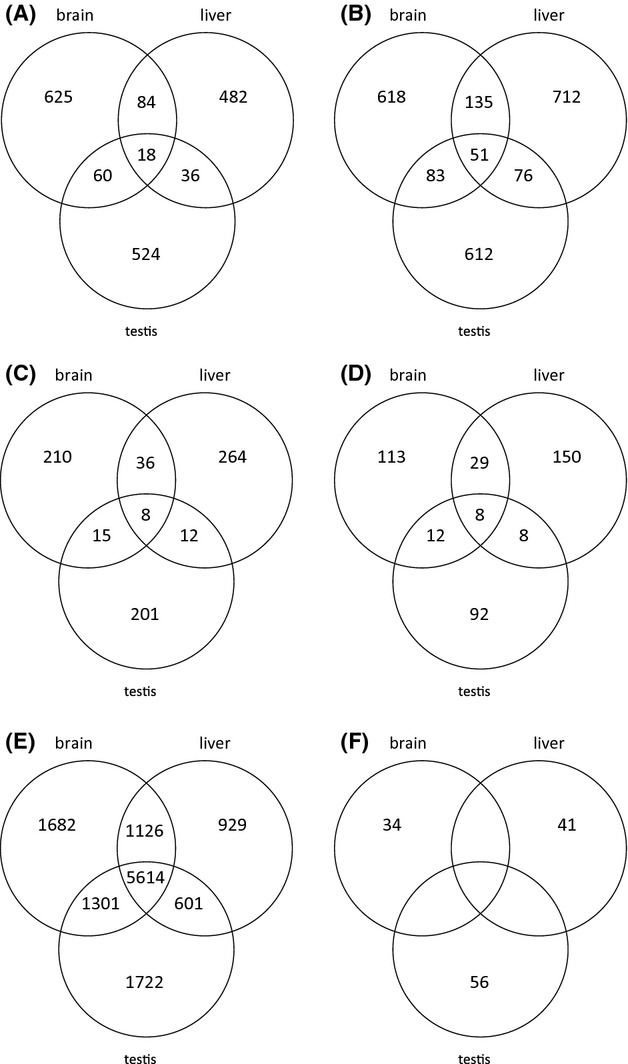
Number of genes differentially expressed between French and German population in different tissues and the overlap of genes between different tissues for A) Affymetrix B) Agilent C) combined set of genes. D) Same as for C, but for genes expressed in all three tissues. E) Number of genes expressed in different tissues expressed in both platforms. F) Number of genes expressed in a single tissue only in both platforms.

Distribution of all genes from the common set among the tissues indicate that testis has significantly more tissue–specific genes than brain and liver, and that brain also has significantly more tissue–specific genes than liver (*n* of all genes expressed in a single tissue in both platforms = 4333; one–sided binomial test: testis > brain *P* < 3.8 × 10^−4^, testis > liver *P* < 4.8 × 10^−97^ and brain > liver *P* < 1.1 × 10^−96^) (Fig. 3E). When we checked the distribution of differently expressed genes among the tissues, we found that liver and testis had significantly more tissue–specific genes than brain (*n* of all genes differently expressed between populations and expressed in a single tissue in both platforms = 131; one–sided binomial test: brain > liver *P* = 0.56, testis > brain *P* = 2.2 × 10^−6^ and testis > liver *P* = 3.3 × 10^−6^) (Fig. 3F). Moreover, 210 of 269 (78%) differently expressed genes in brain, 264 of 320 (83%) in liver and 201 of 236 (85%) in testis are differently expressed only in these single tissues (Fig. 3C). This pattern was also observed in each of the platforms separately (Fig. 3A and B) and for the relaxed cut-off *P*-values of *P* < 0.05 and *P* < 0.1 (data not shown). As this phenomenon could be due to the fact that differently expressed genes are only expressed in a single tissue, we checked whether this pattern held for the 5614 genes expressed in all three tissues. We found that 113 of 162 (70%) differently expressed genes in brain, 150 of 195 (77%) in liver, and 92 of 120 (77%) in testis were also differently expressed only in these single tissues (Fig. 3D).

**Figure 4:**
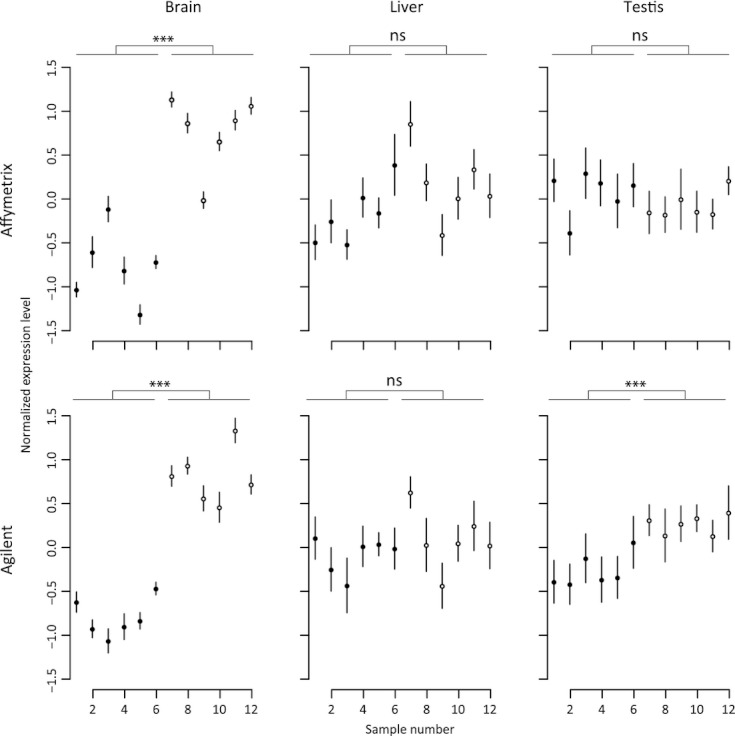
Hierarchical clustering of mean normalized expression levels for 113 genes expressed in all tissues and differentially expressed only in brain in each sample. Columns for liver and testis (both for *n* = 15 genes in both platforms) show mean normalized expression level of these genes in each sample in the respective tissue. Filled circles (samples 1–6): German population; empty cicrles (samples 7–12): French population. Error bars are standard error of the mean. Significance values (two–sided *t*-test without assuming equal variance, comparing mean normalized expression levels between the populations): pAffymetrixBrain = 7.37 × 10^−5^, pAffymetrixLiver = 0.16, pAffymetrixTestis=0.25; pAgilentBrain = 2.75 × 10^−6^, pAgilentLiver = 0.31, pAgilentTestis = 3.7 × 10^−4^. Please note that the pAgilent Testis was significant only in the presented case. Clustering calculations performed for liver and testis otherwise produced nonsignificant values.

However, as expression levels could change in the same direction between populations, but pass the significance cut-off in just one tissue, setting a cut-off value may mask the shared changes between the tissues. We addressed this possibility by performing a hierarchical clustering analysis on expression levels in the brain for the 113 genes that were expressed in all tissues, but differently expressed only in brain. We then selected a cluster (with *n* = 15 genes) with the biggest mean expression difference between the populations on both platforms and calculated mean expression levels of these genes in each sample in the other two tissues. If the shared expression pattern was present among the tissues, these genes should also produce similar separation of the populations in liver and testis. However, we observed very little overlap between brain and the other tissues, indicating that gene expression differences are indeed mostly tissue-specific and not an artifact of relying on a cut-off to select differentially expressed genes (Fig. 4).

### Functional annotation of differentially expressed genes

To assess functional categories of the genes differing between the two populations, we tested whether they were over- or underrepresented in Gene Ontology (GO) categories, KEGG pathways, families of transcription factor (TF) binding sites and protein domain families compared to the common set of expressed genes. Using the GOstats package (Falcon and Gentleman 2007) and GeneTrail software (Keller et al. 2008) (see Methods), we found significant overrepresentation of genes that belong to GO categories and protein domain families in each of the tissues (for the GOstats, we only considered GO categories containing more than five genes; conditional hypergeometric test, *P* < 0.01 after multiple testing correction using Benjamini–Hochberg's FDR; for GeneTrail, we used *P*-value cutoff of 0.05 after multiple testing correction using Benjamini–Hochberg's FDR). We used GOstats package to test Gene Ontology categories rather than GeneTrail, as GOstats allows for correction of inherited annotations of Gene Ontology terms (Falcon and Gentleman 2007). Results from GOstats and GeneTrail analyses are shown in Supplementary Table 2 and 3, respectively.

In the brain, 10 of 30 Biological Process categories with significant overrepresentation of differently expressed genes involve regulation of transcription and other cellular processes. The top three out of six Molecular Function categories enriched in differentially expressed genes in the brain involve transcription factor activity. Zinc-finger and KRAB box protein domains, among others, are enriched among the proteins encoded by the differently expressed genes, and we also find an overrepresentation of genes with binding sites for eight TRANSFAC categories of transcription factors, such as T01108 (CREB-1) and T05114 (SRF), some of which are a part of the MAP kinase signaling pathway (KEGG 04010) that is itself significantly enriched in differentially expressed genes between the populations. Interestingly, none of the above categories are enriched among differentially expressed genes in liver and testis. While there are three other TRANFAC categories of transcription factors and their binding sites enriched in liver, there are none in testis. Instead, we find genes involved in an assortment of phagocytic and proteolytic processes enriched in liver and testis, respectively, as well as in immune-system functions for the genes differentially expressed in liver.

Out of the 13 genes that are differentially expressed between the populations and enriched in the TRANSFAC categories mentioned above, four show higher expression in the German population and they all belong to the TRANSFAC categories T01923 (NF–κB) and T00087 (CBF-A). However, the difference between number of genes showing higher expression in the German population versus the French is not significant (4 vs 9, binomial test *P* < 0.27). Similarly, among the 16 genes encoding proteins with the Zinc- finger and KRAB box domains, four show higher expression in the German population (and three of the proteins have KRAB box domain), but this number is not significantly different from the number of genes showing higher expression in the French population (4 vs 12, binomial test *P* < 0.08).

## Discussion

### Technical considerations

Before going into a closer discussion of the findings with respect to differences between tissues and gene categories, we would like to address the technical issue of data generation via microarray hybridization. Although it is well known that many details of microarray hybridization kinetics are not yet understood (Pozhitkov et al. 2007), it has become customary to accept that such data can be “noisy” and use them for statistical analyses anyway. Still, it is generally recommended that specific results obtained by microarrays should be confirmed using an independent method, ideally quantitative reverse transcription PCR (RT-qPCR). We have performed this in a previous study (Staubach et al. 2010) and found indeed that only about half of the genes found to be differentially expressed in a microarray experiment could be confirmed using RT-qPCR. On the other hand, large–scale confirmation of expression differences by RT-qPCR is not feasible. Hence, we used here two technically different microarray platforms to corroborate the findings. Affymetrix arrays combine the information derived from a set of short probes (21mers) covering a given transcript, whereas Agilent relies on single long probes (60mers). Short probes are inherently much less reliable (Pozhitkov et al. 2007), but the combination of several of them makes up for this deficiency. They may also be more susceptible to polymorphisms, but this is not expected to be a problem in our comparison (see Methods). Therefore, each platform is expected to yield a reasonably reliable result, but we can confirm only less than half of the genes found by each platform alone (Table 1). We assessed whether this is simply due to small differences in *P*-values between the two experiments. However, we find that about a third of the genes that do not fall into the overlap between the two platforms has *P*-values of 0.05 or higher, that is, do not show a comparable response in the other platform. This confirms the notion that the results are much more reliable when they are mutually confirmed by these two independent technical approaches.

### Gene expression evolution in testis

The mouse populations assessed here are derived from a colonization wave of mice into Western Europe that has occurred about 3000 years ago (Cucchi and Vigne 2006). Both populations belong to the same subspecies (*M. m. domesticus*), but can be molecularly differentiated and our previous analysis based on 186 microsatellites in 60 unrelated individuals from each population indicates no gene flow between them (Ihle et al. 2006). Giving the relatively recent divergence time, we find it rather noteworthy that we observe quite a number of loci with significant changes in expression. This supports the notion that gene expression diverges fast, although most of this is likely to be due to neutral divergence (Khaitovich et al. 2004; Staubach et al. 2010). Indeed, while we are unable to tell whether the changes we observe are due to directional selection or neutral, we note that in a recent experiment in our group that estimated amount and location of selective sweeps in several populations on wild mice (including the ones used in this study) using whole-genome SNP data, the overlap between our differentially expressed genes and genes under selective sweep there was less than five percent (Staubach et al. 2012), suggesting that the divergence in gene expression that we report is to a large extent driven by nonadaptive processes.

**Figure 5:**
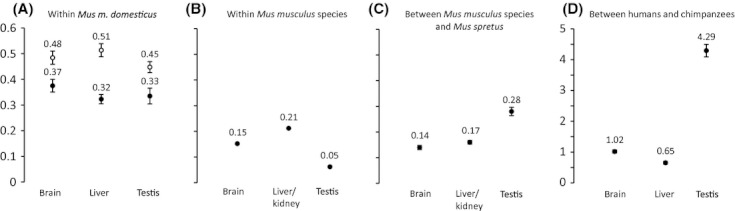
Mean scaled divergence and 95% confidence intervals in gene expression in different tissues in groups with different level of divergence. A) German versus French population of *Mus musculus domesticus* based on Affymetrix (filled circles) or Agilent (empty circles) data; divergence time less than 3000 years (Cucchi and Vigne 2006; Bonhomme et al. 2011) B) Average between pairwise comparisons of *Mus m. musculus*, *Mus m. domesticus*, and *Mus m. castaneus*, diverged 500,000–800,000 years ago (Guénet & Bonhomme 2003) C) between *Mus spretus* and average from all pairwise comparisons of *Mus musculus* subspecies; diverged 1–1.5 Mya (Boursot et al. 1993) D) humans versus chimpanzees, diverged 5–7 Mya (Patterson et al. 2006). Note that panel D is plotted on a different scale. Panels B and C are from (Voolstra et al. 2007) (data kindly provided by Bettina Harr and Christian Voolstra), panel D is based on data from (Khaitovich et al. 2005) provided by Mehmet Somel.

Our analysis qualitatively confirms the finding of Voolstra et al. (2007) that the testis gene divergence within species is not faster than that of the other tissues. To illustrate the pattern of gene expression divergence across populations and species, we plotted data from this study (comparison within *Mus musculus domesticus* populations) alongside data from Voolstra et al. (data within *Mus musculus* subspecies and between *Mus musculus* and *Mus spretus* species) and from Khaitovich et al. (data for Homo sapiens and Pan troglodytes) (Fig. 5). While testis gene expression divergence is low between *Mus musculus* populations and subspecies, it increases in between species comparisons in Mus, humans and chimpanzees. However, the lack of testis divergence in early stages of population differentiation is superficial, as we show here that testis gene expression diverges nonetheless in a different way from other tissues. In our measures of correlation of t statistics between the tissues, testis was least correlated with the other tissues, whereas brain and liver were highly correlated. The observations that testis divergence is qualitatively different from the other tissues, and that we cannot associate differently expressed genes in testis with specific transcription factor targets, suggests that short-term divergence in this tissue may be largely driven by alternative mechanisms, i.e. changes in the concentrations of transacting factors like microRNAs, or chromatin modifier proteins, may be more relevant for testis-specific cis-acting factors.

### Enhancer-specific divergence

We also observed that the majority of genes that are differently expressed between the two populations are differently expressed only in a single tissue, even if these genes are expressed in all tissues tested. This implies tissue-specific factors or enhancers diverging independently of each other and is consistent with previous studies performed on different mouse species (Voolstra et al. 2007; Staubach et al. 2010) and on primates (Blekhman et al. 2008), suggesting a general pattern of flexibility of gene expression regulation in specific tissues over large evolutionary time span. Hence, this confirms the notion that different tissue-specific enhancers of a gene are decoupled in an evolutionary sense: they may each independently be targets of drift or selection.

### Transcription factors have a role in early brain divergence

Perhaps the most intriguing result of functional classification of differently expressed genes is the overrepresentation of genes regulated by several important transcription factors among differently expressed genes in brain and liver. This phenomenon was observed before in primates (Gilad et al. 2006) and recently between humans and chimpanzees (Nowick et al. 2009). In the latter comparison, the family of KRAB transcription factor protein domains that is overrepresented in our comparison is also overrepresented in human brain compared to chimpanzee. Overall, this finding suggests that changes in gene regulation driven by TF may be a general pattern in diverging brains and to a smaller extent in livers, irrespective of evolutionary distance between the compared groups. It also suggests that it may not necessarily be implicated in differences in cognitive abilities (Gilad et al. 2006; Nowick et al. 2009), although we cannot exclude the possibility that the two mouse populations investigated in this study differ in this respect.

## Methods

### Animal capture and tissue collection

We bred F1 offspring of wild-caught mice from each of the two populations in the laboratory. Parental mice were caught in the Massif Central in France and in the Cologne/Bonn area in Germany (see Results and (Ihle et al. 2006)). Six unrelated males aged 12 weeks were sacrificed using CO2. Their organs were excised, immediately snap frozen in liquid nitrogen, and stored at −80°C. All mice were dissected at the same time of the day. All animal work was registered under number V312-72241.123-34 (97-8/07) and approved by the ethics commission of the Ministerium für Landwirtschaft, Umwelt und ländliche Räume on 27.12.2007.

### Sample preparation and microarray runs

RNA was isolated from tissues using Trizol® (Invitrogen, Life Technologies Corporation, Carlsbad, CA) following the manufacturer's protocol and its quality assayed using Agilent Technologies 2100 Bioanalyzer and the RNA 6000Nano LabChip kit (Agilent Technologies, Santa Clara, CA). Using the Mouse Genome 430 2.0 GeneChip (Affymetrix, Santa Clara, CA), we determined expression profiles for 39,000 mouse transcripts in all 12 mice of the respective populations per tissue (36 samples in total; no technical replicates were run). The same 36 RNA samples (no technical replicates were run), were hybridized to Agilent Mouse Genome 4 × 44k arrays and scanned on Agilent G2505C scanner (Agilent Technologies). Single-color labeling, hybridization, and scanning on both platforms were performed using standard manufacturers' protocols.

#### Data analysis

All analyses were performed using R and Bioconductor (Gentleman et al. 2004, 2005).

#### Affymetrix data

Affymetrix data were checked for possible differences in probe binding affinity due to sequence differences between the reference genome and the genome of *Mus musculus domesticus* using R package mask (Dannemann et al. 2009). As only fewer than 0.005% of probes showed differences in binding, no changes were made to the probe content. Affymetrix data were annotated using custom CDF files from Brainarray (Version 12) (Dai et al. 2005), then background corrected and normalized using GCRMA. Data were analyzed on a per tissue basis.

Affymetrix data from each tissue were also tested for potential cage effects or technical artifacts in sample handling by principal component analysis; in all cases, we observed clear groupings of samples according to their population, indicating no overt confounding effects of nonbiological factors.

#### Nonspecific filtering

**Figure 6:**
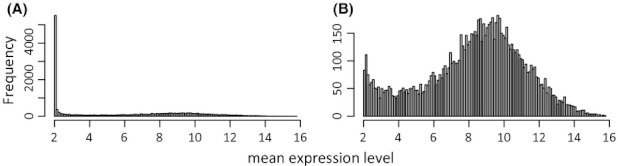
Nonspecific filtering of the Affymetrix data to remove unexpressed and/or uninformative probes from the data. A) Distribution of number of genes and expression levels in the unfiltered dataset in the brain (*n* = 15041 genes) B) The same distribution after removing 5956 genes for which interquartile range was less than shorth of the distribution.

To exclude noninformative signals, genes were filtered according to their variability across all samples from both population: all genes for which variation was less than shorth (the shortest interval containing half the data) of interquartile range were removed from further analyses (Gentleman et al. 2004; Falcon and Gentleman 2007; Hahne et al. 2008); below we detail our rationale for applying it to the data. The distribution of mean normalized expression levels for all genes on Affymetrix platform for brain (which is representative for other tissues as well) indicated a skew toward very low expression values (Fig. 6A). Genes with very low expression levels are associated with high expression variation among individuals, thus making it difficult to detect differential gene expression between populations. However, it is possible that among genes with low expression values there are some that are truly differentially expressed. To remove the noise without sacrificing true differentially expressed genes, we aimed to remove invariant genes, that is, genes which expression value is stable across all samples from both populations combined. We have sought to arrive at the right filtering procedure by trying variation based on standard deviation (SD) and interquartile range (IQR), and using median or shorth of these distributions as threshold. We found that shorth of the IQR-based threshold to be acceptable (Fig. 6B): in the brain this filtering removed 5956 out of 15041 genes (40%), and for only four of the removed genes there were significant differences in expression between the two populations (*P* < 0.05 nonmoderated *t*-test assuming equal variances and not correcting for multiple testing; there were none when *P* < 0.01 was used), indicating that variation-based filtering is extremely efficient in removing uninformative signal from data. In addition, when we run the moderated t-test on the whole unfiltered dataset, we found almost exactly the same number of differentially expressed genes as in the filtered dataset (696 vs 691), further indicating that the filtering procedure does not negatively affect the number of detected differentially expressed genes. It does, however, positively affect *P*-values: in the unfiltered dataset *P*-values for 85 genes are higher enough compared with the filtered dataset that they would not be identified as differentially expressed with our *P* < 0.01 threshold (the *P*-values for these genes are >0.01 and <0.05).

## Agilent data

The Agilent data were background corrected using normexp algorithm and normalized using quantile normalization with MeanSignal and BGUsed as the proper and background signals, respectively, with offset of 10, as implemented in limma (Smyth 2004) and Agi4 × 44PreProcess packages. Only probes whose signals were identified on at least three samples were retained. To construct a common set of genes interrogated by the two platforms, only the Agilent probes that were annotated to genes included in the Affymetrix analysis, as determined using BioMart database, were retained. In cases where multiple Agilent probes were annotated to a gene from the Affymetrix analysis, only the Agilent probe with the lowest *P*-value from a moderated t-test per gene was retained, as it represented the most differentially expressed transcript and as such was most informative for this experiment.

## Combined dataset

Finally, differentially expressed genes (Storey and Tibshirani 2003) were identified using a moderated t-test as implemented in limma package and Mann–Whitney *U-*test. Gene Ontology analysis was performed using GOstats (Falcon and Gentleman 2007) with the following settings: *P*–value cutoff 0.01 in the conditional hypergeometric test for overrepresentation of genes in a category with minimum five genes in a category and GeneTrail (Keller et al. 2008) with the following settings: adjustments for multiple testing using Benjamini & Hochberg FDR and 0.05 as the significance threshold after adjustment.

The neighbor joining trees were based on Euclidean distances between sample pairs, calculated using standard normalized data (each gene's expression level has mean = 0 and standard deviation = 1). We used the R ape package (Paradis et al. 2004) for calculating unrooted trees and stats package to classify genes using unsupervised hierarchical clustering.

## Data Accessibility

Gene expression data from this study has been deposited in EBI's ArrayExpress according to MIAME guidelines under the submission number E-MEXP-3337.

## Supporting Information

**Table S1.** Common set of genes with expression values, corresponding P values, t statistics values and related parameters for each platform.

**Table S2.** Results from the GOstats analysis.

**Table S3.** Results from the GeneTrail analysis.
